# Home-Based Electronic Cognitive Therapy in Patients With Alzheimer Disease: Feasibility Randomized Controlled Trial

**DOI:** 10.2196/34450

**Published:** 2022-09-12

**Authors:** Anna Marin, Renée DeCaro, Kylie Schiloski, Ala’a Elshaar, Brigid Dwyer, Ana Vives-Rodriguez, Rocco Palumbo, Katherine Turk, Andrew Budson

**Affiliations:** 1 Center for Translational Cognitive Neuroscience Veterans Affairs Boston Healthcare System Boston, MA United States; 2 Department of Neurology Boston University School of Medicine Boston, MA United States; 3 Alzheimer's Disease Research Center Boston University School of Medicine Boston, MA United States

**Keywords:** cognitive training, Alzheimer disease dementia, technology

## Abstract

**Background:**

Can home-based computerized cognitive training programs be a useful tool to sustain cognition and quality of life in patients with Alzheimer disease (AD)? To date, the progressive nature of the disease has made this question difficult to answer. Computerized platforms provide more accessibility to cognitive trainings; however, the feasibility of long-term, home-based computerized programs for patients with AD dementia remains unclear.

**Objective:**

We aimed to investigate the feasibility of a 24-week home-based intervention program using the Constant Therapy app and its preliminary efficacy on cognition in patients with AD. Constant Therapy is a program developed for patients with speech and cognitive deficits. We hypothesized that patients with AD would use Constant Therapy daily over the course of the 24-week period.

**Methods:**

Data were collected over a 48-week period. We recruited participants aged between 50 and 90 years with a diagnosis of mild cognitive impairment due to AD or mild AD dementia. Participants were randomly assigned to either the Constant Therapy (n=10) or active control (n=9) group. The Constant Therapy group completed a tablet-based training during the first 24 weeks; the second 24 weeks of computerized training were optional. The active control group completed paper-and-pencil games during the first 24 weeks and were invited to complete an optional Constant Therapy training during the second 24 weeks. Every 6 weeks, the participants completed the Repeatable Battery for the Assessment of Neuropsychological Status (RBANS). The participants independently accessed Constant Therapy using an Apple iPad. Our primary feasibility outcomes were the rate of adherence and daily use of Constant Therapy over 24 weeks. Our secondary outcomes were Constant Therapy performance over 24 weeks and change in RBANS scores between the 2 experimental groups.

**Results:**

Feasibility analyses were computed for participants who completed 24 weeks of Constant Therapy. We found that long-term use of the Constant Therapy program was feasible in patients with AD over 24 weeks (adherence 80%; program use 121/168 days, for 32 minutes daily). These participants showed an overall improvement in accuracy and latency (*P*=.005) in the Constant Therapy scores, as well as specific improvements in visual and auditory memory, attention, and arithmetic tasks. The Constant Therapy group showed improvement in the RBANS coding subtest. No unexpected problems or adverse events were observed.

**Conclusions:**

Long-term (eg, 24 weeks) computerized cognitive training using Constant Therapy is feasible in patients with AD in the mild cognitive impairment and mild dementia stages. Patients adhered more to Constant Therapy than to the paper-and-pencil training over 24 weeks and improved their performance over time. These findings support the development of future randomized controlled trials that will investigate the efficacy of Constant Therapy to sustain cognitive function in patients with AD.

**Trial Registration:**

ClinicalTrials.gov NCT02521558; https://clinicaltrials.gov/ct2/show/NCT02521558

## Introduction

### Background

A total of 5.8 million people aged ≥65 years are living with Alzheimer disease (AD) dementia in the United States [1], highlighting a need for effective long-term cognitive interventions for these people. Cholinesterase inhibitors can help turn the clock back on the disease 6 to 12 months [2]. Aducanumab may possibly slow disease progression slightly, equivalent to 3 months, in patients with mild cognitive impairment (MCI) due to AD and mild AD dementia [3]. However, medications alone cannot halt the disease, and supplementing pharmacological interventions with nonpharmacological interventions has been shown to sustain cognition and quality of life more than medication alone [4-6].

Cognitive training programs are a traditional nonpharmacological intervention consisting of guided practice on standardized tasks to enhance specific cognitive functions, which may ultimately aid cognition and daily functioning [7]. During a typical session, patients complete tasks of varying difficulty, targeting different cognitive domains, such as memory, attention, and problem solving. The training is completed individually or in group sessions using paper and pencil or computerized programs under the supervision of a clinician. The repeated practice of tasks over time is aimed at improving or sustaining cognitive performance [7]. For example, patients with mild AD dementia showed improvements in their Mini-Mental State Examination scores when medication treatments were supplemented with a year of one-on-one regular cognitive training for 5 days weekly [5]. Supervised cognitive training has also been found to be beneficial in older adults with memory loss due to AD or vascular dementia by enhancing cognitive functioning and well-being in daily life [7-10]. Furthermore, a previous study of healthy older adults found that cognitive benefits were preserved 5 years after cognitive training [11]. Despite the potential benefits, traditional cognitive training programs require face-to-face contact, are expensive (staff prices from US $15 to US $100 per hour), and demand a significant time commitment (at least 60 minutes daily for 3 weeks) for the patient to make any gains. Therefore, it is challenging for patients with AD to adhere to traditional cognitive training programs [12].

Home-based, self-administered computerized cognitive training represents a practical alternative to overcome the expense and adherence challenges seen in traditional supervised-in-person cognitive training programs. Computerized cognitive training allows individuals to independently access cognitive exercises from their own computers, tablets, or other mobile devices at any time [13]. Home-based self-administered computerized cognitive training has been shown to benefit cognitive function as much as supervised-in-person training sessions in healthy older adults [12,14]. However, computerized cognitive training has produced mixed results in patients with AD. Some studies show positive effects, others show a temporary effect or protection from decline, and some show no effect [15,16]. Two possible explanations for these discrepant findings could be the variability in the duration and level of difficulty of the training program. The progressive nature of AD-related cognitive decline also adds to the difficulty in accurately testing the effectiveness of home-based computerized programs. Many investigators endorse the need for research assessing the effects of longer and more individualized intervention programs that can adjust the level of task difficulty depending on the baseline cognitive function of patients [16-19].

Given the influence of factors such as age and clinical diagnosis on the effectiveness of computerized cognitive training on cognition [20], several platforms have been developed to provide more flexibility and accessibility for older adults [21], patients with AD [22], and other neurological diagnoses (eg, stroke, traumatic brain injury [TBI], and schizophrenia) [23].

We assessed the feasibility and preliminary efficacy of a 24-week individualized computerized program called Constant Therapy. Constant Therapy is a digitally delivered, cloud-based computerized training program developed for patients with speech and cognitive deficits. During a home-based, self-administered Constant Therapy session, patients practice computerized exercises in increasing order of difficulty. As they progress through their intervention schedule, the tasks change their level of difficulty, depending on the patients’ individual progress. Constant Therapy allows patients to practice and advance independently such that patients experiencing different patterns of cognitive impairment can advance through the program at their own pace. Constant Therapy has been successfully implemented in individuals with aphasia due to stroke or TBI, with findings showing, on average, 70% compliance with the Constant Therapy self-administered training over the course of 10 to 20 weeks, improvements in task scores over time, and carryover to standardized assessment measures [24-26]. In these studies, accuracy (correct responses) and latency (reaction time) measures are used to quantify task performance. Increased accuracy and decreased latency characterize improved task performance [24]. The Constant Therapy platform has been studied both in the clinic and home environment. Patients using the platform at home make similar improvements compared with those who use the platform with their clinicians in the clinic [25].

No study has assessed the feasibility of the Constant Therapy app in the AD population. If feasible in the AD population, this type of home-based computerized intervention might have the potential to enhance cognitive functioning and support well-being in the daily lives of patients with AD.

Our study aimed to test the feasibility of long-term computerized cognitive Constant Therapy training (24 weeks) in the AD population.

### Objectives

The primary aim of this study was to assess the feasibility of Constant Therapy in patients with AD using a long-term individualized training program. We measured adherence to the Constant Therapy program over a period of 24 weeks using a randomized design. We hypothesized that patients with AD would use the Constant Therapy app daily and would adhere to the training over a 24-week period [27].

The secondary aim was to evaluate the preliminary efficacy of the training on the performance of Constant Therapy tasks and on standardized assessments of cognition and daily life functioning. As in the previous study by Des Roches et al [24], we assessed task performance (accuracy and latency), as any improvements could relate to willingness to continue adherence to the task and could suggest benefits to cognitive function. We hypothesized that the patients’ performance after the long-term intervention period would be less impaired compared with patients who have not completed the Constant Therapy intervention. We also expected sustained improvement in performance on both the Constant Therapy tasks over 24 and 48 weeks and on neuropsychological scores after the intervention.

## Methods

### Study Design

**Figure 1:**
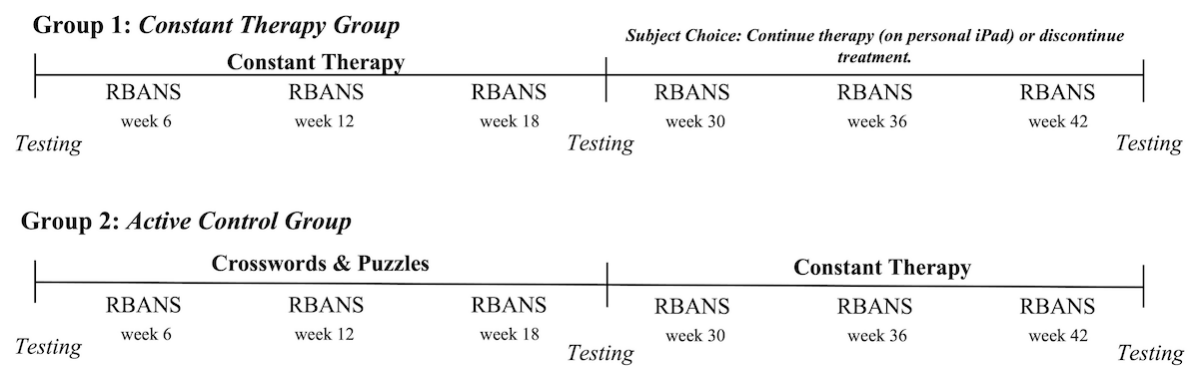
Study design. RBANS: Repeatable Battery for the Assessment of Neuropsychological Status.

The study was an unblinded randomized controlled trial (RCT) in which every newly recruited participant was randomly assigned to either training condition for 24 weeks. Participants’ condition assignment was completed by the study coordinator using a web-based random number generator. Data were collected between October 2016 and January 2019. At the end of the 24 weeks, participants randomly assigned to the Constant Therapy training condition were allowed to continue using the app or to discontinue, while those in the active control training condition were offered the opportunity to use the app (48 weeks total; Figure 1).

### Ethics Approval

All data from the patients’ devices were anonymized during collection. This project was conducted under the Boston University Medical Center Institutional Review Board under study protocol H-34203.

### Participants

Patients were referred by the Boston University Alzheimer’s Research Center or by practicing neuropsychologists, neurologists, or other associated discipline from Boston Medical Center. After referral, potential participants were contacted by a member of our research staff, who described the details of the study. We enrolled participants between 50 and 90 years of age that had a diagnosis of MCI due to AD or mild AD dementia. The diagnosis was confirmed by the referring clinician following the National Institutes of Aging-Alzheimer’s Association criteria [28,29]. Moreover, the baseline cognitive battery was administered to all participants before beginning either training type to evaluate cognitive function at baseline and to ensure that the level of impairment did not exceed the mild AD dementia status.

Patients with any self-reported history of substance abuse, prior head trauma (eg, stroke or TBI), significant depression, or other mood disorders were not considered eligible to participate in the study. Referring clinicians evaluated the exclusion of the patient from the study based on clinical notes and self-report.

### Study Training and Baseline Cognitive Testing

Before starting the intervention phase, participants were trained on how to use the Constant Therapy app and how to navigate an Apple iPad, such as how to access the app, switch between tasks, and complete each individual task. iPads were loaned to participants if they did not already own one. The cognitive testing and app training at the start of the study were done across 2 days for approximately 1 to 2 hours each day to avoid exhausting the participants. The research staff completed the 2-day preintervention training either from the laboratory site or in the participant’s house, depending on the participant’s preference. During the first day of training, the research staff completed baseline cognitive testing; on the second day, they completed an overview of the Constant Therapy platform and iPad (if assigned to the Constant Therapy condition) or an overview of the booklets containing the crosswords and puzzle (if assigned to the active control condition).

The baseline cognitive battery consisted of the Montreal Cognitive Assessment [30], the Repeatable Battery for the Assessment of Neuropsychological Status (RBANS), the Multifactorial Memory Questionnaire [31], an Activities of Daily Living Scale [32], a Quality of Life in Alzheimer’s Disease Scale [33], the Neuropsychological Assessment Battery for Memory [34], the Zarit Burden Interview [35], and the False Memory Questionnaire [36]. Following the initial neuropsychological assessment, the patients were randomly assigned to 1 of 2 groups (Figure 1). The same battery was repeated after 24 and 48 weeks of participation in the study to monitor changes in cognition and functioning in daily life throughout the study. To ensure consistency between testing sessions, the same research staff member completed assessments at month 0, month 24, and month 48. Testing sessions were conducted in the laboratory or at the participant’s home. The RBANS was also administered at weeks 6, 12, 18, 30, 36, and 42 by the same assessor.

Research staff remotely monitored individual participant progress on the Constant Therapy platform daily. The study staff called the participants weekly to keep reminders consistent between both intervention groups. During these calls, the staff asked about adherence to the tasks, answered any questions, and reminded individuals to engage with the platform.

The research members completing the baseline, interim, and final assessments were not blinded to the participants’ group assignment to ensure that participants could reach out for support and questions throughout the duration of the study.

### Intervention

#### Constant Therapy Group

Patients in the Constant Therapy group (group 1, Figure 1) received the Constant Therapy program for a planned 24 weeks. Progress on the tasks was monitored daily, and weekly phone calls were completed to check in with participants. Participants were instructed to engage in Constant Therapy for approximately 30 minutes a day. The software recorded the amount of time spent performing the cognitive tasks. The neuropsychological testing battery performed at the start of the study was repeated at the end of the first 24 weeks. At this stage, participants were offered the option to either continue with the Constant Therapy training for an additional 24 weeks or to terminate their participation in the study. At the end of 48 weeks, the testing battery performed at the start of the study was repeated.

#### Active Control Group

During the first 24 weeks of the study, participants in the active control group (group 2, Figure 1) received booklets containing different types of puzzles and brain teasers (crossword puzzles, word search puzzles, Sudoku puzzles, and various types of math puzzles). They were instructed to perform these tasks for approximately 30 to 60 minutes per day. We monitored adherence to these puzzles weekly via phone conversations, mirroring the Constant Therapy group. After 24 weeks and the completion of the testing battery, the active control group was invited to participate in the Constant Therapy training for the following 24 weeks. The testing battery of neuropsychological assessments was completed at weeks 0, 24, and 48, as in the Constant Therapy group.

### Constant Therapy Training Program

Data were collected using the Constant Therapy app, which includes evidence-based speech, language, and cognitive exercises with varying levels of difficulty ranging from level 1 to level 10 (the software can be reviewed and accessed through the web link [37]). A total of 3 scores of 80% or higher advanced participants to the next difficulty level of a task. The tasks were designed with the aim of improving or stabilizing language, attention, and memory functioning. The exercises tested domains of language (naming, comprehension, speaking, reading, and writing) and cognitive skills (attention, executive skills and problem solving, mental flexibility, memory, and visuospatial skills). The Constant Therapy program recorded performance data (task accuracy and latency) as well as all other session activities (usability logs, use of built-in cues within the app, time stamps, and item completion indicators). For a more detailed review of the platform and cognitive tasks, refer to the study by Kiran et al [38].

In this pilot study, we examined the feasibility of Constant Therapy tasks in patients with AD, with the future goal of testing in a larger RCT how Constant Therapy tasks could help stabilize or improve some of the cognitive domains most prominently affected by AD (eg, memory, language, and executive function) [39,40]. All tasks were self-paced and self-administered by the participants. All tasks gave the participants the option to skip or quit at any time if they felt fatigued or frustrated. The Constant Therapy tasks used in this study were not modified for the population with AD.

### Outcome Measures

We collected primary feasibility measures and secondary preliminary efficacy measures (Textbox 1).

**Outcome measures.**

**Outcome measures**

1. Primary feasibility measures
  Overall adherence to the study design up to 24 weeks
  Adherence rates in the Constant Therapy and the active control training during the first 24 weeks
  Use of the Constant Therapy app over the first 24 weeks
  Any engagement with the app during voluntary continuation to 48 weeks
2. Secondary preliminary efficacy variables
  Constant Therapy tasks performance (accuracy and latency):
    Arithmetic tasks (addition, multiplication, subtraction, and division)
    Auditory tasks (environmental sound matching, spoken word matching, voicemail, and auditory command)
    Visual tasks (calendar reading, clock math, clock reading, map reading, mental rotation, pattern recreation, picture matching, face matching, picture n-back memory, playing-card slapjack, symbol matching, written word matching, and flanker)
    Quantitative reasoning tasks (currency, functional math, number pattern, and word problem)
  Repeatable Battery for the Assessment of Neuropsychological Status subtest scores at week 0, 6, 12, 18, and 24:
    Memory: list learning, list recall, list recognition, story immediate recall, story, and delayed recall
    Language: picture naming and semantic fluency
    Executive function: digit span and coding
    Visuospatial and constructional: figure copy and line orientation

### Statistical Analysis

#### Sample Size

Prior work has studied the feasibility and preliminary efficacy of computerized cognitive training over 12 weeks in patients with MCI and AD dementia using RCTs with sample sizes of 11 [41], 20 [42], and 22 participants [43].

#### Analytic Plan

The overall data analyses conducted aimed to primarily assess the feasibility of the Constant Therapy program in patients with AD over the course of 24 and 48 weeks, with secondary analyses examining the preliminary efficacy of the Constant Therapy program for improving or stabilizing cognitive function over an extended period.

#### Demographics

A total of 19 participants (18, 85% male and 1, 5% female) aged 64 to 85 years from the Boston University Alzheimer’s Disease Research Center and Boston Medical Center were enrolled in the study and were randomly assigned to an experimental condition. They met the criteria for MCI due to AD (n=7, 37%) or mild AD dementia (n=12, 63%), as described by the National Institutes of Aging-Alzheimer’s Association criteria [28,29]. All study participants were non-Latino White people.

The demographics of the 19 participants were analyzed using descriptive statistics and included age, education (years of schooling), and baseline scores of cognition and daily life functioning. We used the Mann-Whitney Wilcoxon test to evaluate differences between the Constant Therapy and active control group.

#### Study Adherence

We measured the rate of adherence to this novel long-term intervention program lasting between 24 and 48 weeks. Adherence rate was calculated by counting the number of participants enrolled in the program every 6 weeks. As individuals were given the option to continue after 24 weeks, we also reported the rate of adherence up to 48 weeks.

#### Constant Therapy Usage

We then performed an analysis of engagement to the Constant Therapy program by computing the average number of days each participant spent on the app over the course of the intervention, as well as the average time spent on the app daily.

#### Constant Therapy Tasks Performance

To assess our secondary aim, we analyzed the change in performance on the Constant Therapy tasks measured by accuracy and latency scores for each task. We conducted a Wilcoxon signed-rank test to determine changes in performance (accuracy and latency) for each task. We compared scores for each task at the start (average of the first 10 trials) and at the end (average of the last 10 trials) of the 24 weeks of training. The first 3 observations were excluded before averaging the 10 initial scores to account for the practice time required to adapt to the Constant Therapy platform [24].

#### Preliminary Efficacy of Constant Therapy on Clinical Variables

Our ability to compare changes in cognitive performance between week 0 and week 24 was limited. The RBANS was the only measure that was repeated 5 times throughout the 24-week period (baseline, week 6, week 12, week 18, and week 24). The rest of the outcome measures (Montreal Cognitive Assessment, Zarit Burden Interview, Multifactorial Memory Questionnaire, Activities of Daily Living Scale, Quality of Life in Alzheimer’s Disease Scale, False Memory Questionnaire, and Neuropsychological Assessment Battery) were administered twice (baseline and week 24) and could not be further analyzed due to a higher dropout rate in the active control group than in the Constant Therapy group. Thus, we conducted an exploratory analysis using only the RBANS subtest scores to evaluate the preliminary efficacy of the Constant Therapy training. None of the participants dropped out from week 0 to week 6; therefore, we assessed the changes in performance over the first 6 weeks between the Constant Therapy and active control groups. While normality was not always violated (only in 4 out of 12 RBANS submeasures), we computed a Mann-Whitney test to protect against type 1 and type 2 errors that are likely to occur with our small sample size. To further explore the feasibility of the training, for the RBANS subtest that were normally distributed, we performed a 2×5 repeated measures ANOVA with the factors of group (2: Constant Therapy vs active control) and time (5: week 0, 6, 12, 18, and 24). This was tested for group differences at each time point. Post hoc comparisons were performed using the Tukey honestly significant difference test.

## Results

### Demographics

The Constant Therapy and active control groups did not differ in demographic variables, as determined using the Mann-Whitney Wilcoxon test (Table 1).

**Table 1:** Demographics at baseline test.

Characteristics		Constant Therapy (n=10)	Active control (n=9)	*P* value
Sex (male), n (%)		10 (100)	8 (89)	N/A^a^
**Diagnosis, n (%)**				
	MCI^b^ due to AD^c^	4 (40)	3 (33)	N/A
	Mild AD dementia	6 (60)	6 (67)	N/A
Ethnicity and race (non-Latino White)		10 (100)	9 (100)	N/A
Age (years), median (IQR)		72.50 (14.00)^d^	75.00 (13.00)	.78
Education, median (IQR)		14.00 (4.00)	14.00 (5.50)	.91
**Outcome measures, median (IQR)**				
	MoCA^e^	20.50 (6.00)^d^	21.50 (2.00)^d^	.97
	ZBI^f^	13.00 (15.25)	6.00 (10.00)^d^	.97
	MMQ^g^	103.00 (26.75)^d^	129.00 (29.00)^d^	.10
	ADL^h^	12.50 (22.75)	26.00 (39.00)^d^	.50
	QOL-AD^i^	37.50 (19.75)^d^	36.00 (14.00)^d^	.91
	FMQ^j^	43.50 (45.75)^d^	56.00 (47.00)^d^	.84
**RBANS^k^, median (IQR)**				
	Total list learning	20.00 (9.00)^d^	20.50 (8.50)^d^	.66
	List recall	1.00 (3.25)	0.50 (2.75)	.45
	List recognition	15.00 (5.25)^d^	15.00 (4.75)^d^	.91
	Story memory (immediate recall)	14.00 (4.50)	10.50 (4.00)^d^	.11
	Story memory (delayed recall)	3.50 (5.25)^d^	4.00 (4.00)^d^	.72
	Digit span	9.500 (5.50)^d^	10.00 (3.00)^d^	.72
	Figure copy	20.00 (2.00)	19.00 (1.00)	.11
	Figure recall	3.500 (13.50)	4.00 (6.00)	.66
	Semantic fluency	15.00 (10.25)	16.50 (6.75)^d^	.36
	Line orientation	17.00 (5.25)^d^	17.00 (5.25)^d^	.50
	Picture naming	9.50 (1.00)	9.50 (1.00)	.99
	Coding	27.00 (14.75)^d^	32.50 (12.50)^d^	.72

^a^N/A: not applicable.

^b^MCI: mild cognitive impairment.

^c^AD: Alzheimer disease.

^d^Normally distributed.

^e^MoCA: Montreal Cognitive Assessment.

^f^ZBI: Zarit Burden Interview.

^g^MMQ: Multifactorial Memory Questionnaire.

^h^ADL: Activities of Daily Living Scale.

^i^QOL-AD: Quality of Life in Alzheimer’s Disease Scale.

^j^FMQ: False Memory Questionnaire.

^k^RBANS: Repeatable Battery for the Assessment of Neuropsychological Status.

### Study Adherence

#### Constant Therapy Group Adherence

**Figure 2:**
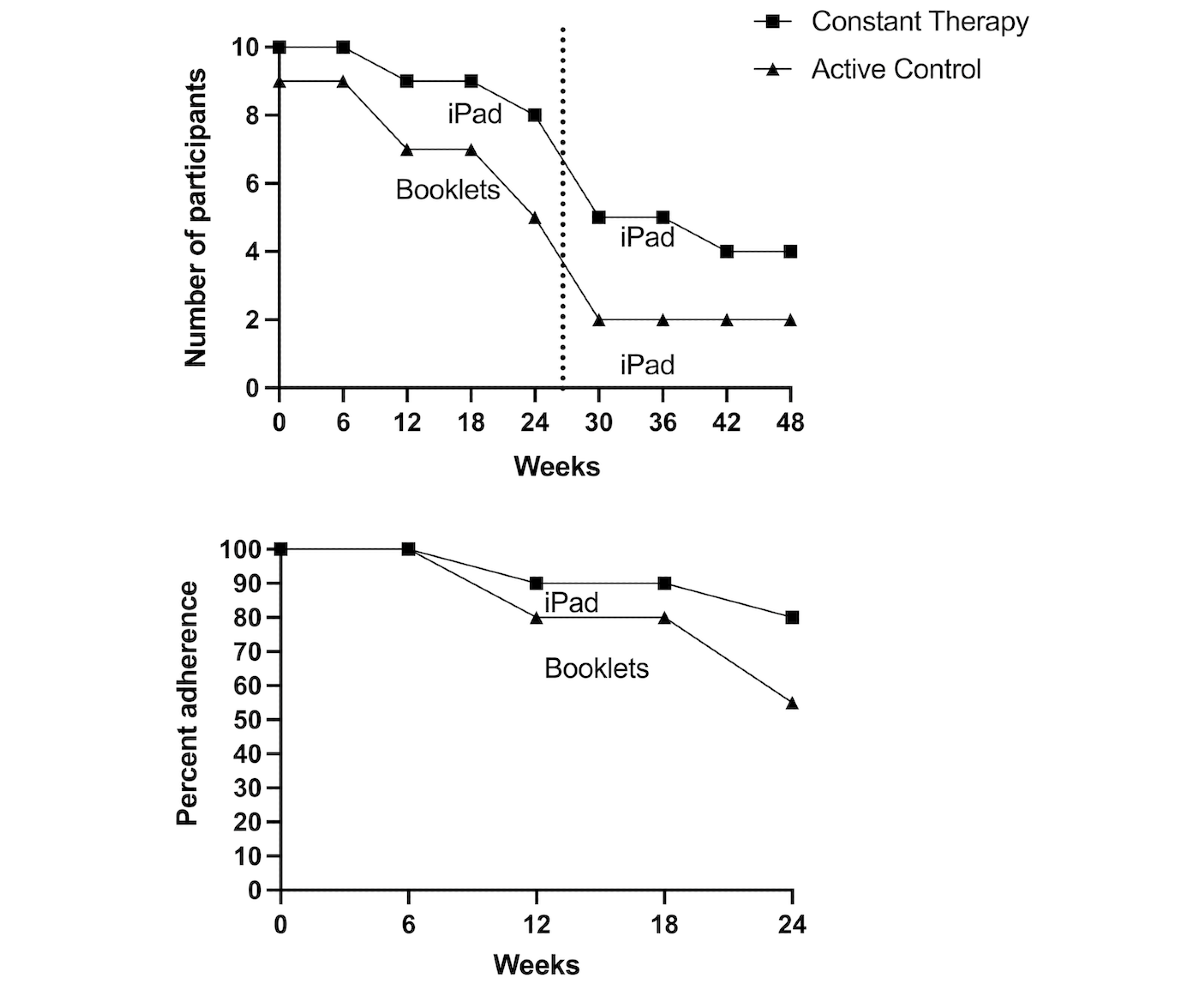
Study adherence.

As shown in Figure 2, 80% (8/10) of participants in the Constant Therapy group completed 24 weeks of the intervention; 5 patients continued the study beyond the 24-week period, 1 to 42 weeks (10%) and 4 to 48 weeks (40%).

#### Active Control Group Adherence

In total, 55% (5/9) of participants in the active control group completed the 24 weeks of the study. Overall, 22% (2/9) of participants elected to engage with the Constant Therapy app until week 48. No unexpected problems were observed in this study. The reasons for dropping out of the intervention were not collected, and individuals who dropped out of the intervention were also discontinued from their postintervention clinical assessment.

The following analysis used data collected between week 0 and week 24.

#### Constant Therapy Use

We examined app use over 24 weeks (168 days) in the Constant Therapy intervention group (Multimedia Appendix 1). On average, participants engaged with the app a mean of 121.4 (SD 38.56, 95% CI 97.50-145.30) days, with 31.70 (SD 9.94, 95% CI 25.54-37.86) minutes spent on the app per day. Participants with MCI due to AD (n=4) spent a mean of 147.50 (SD 21.30, 95% CI 126.63-168.37) days, with 38.16 (SD 11.09, 95% CI 27.29-49.02) minutes per day on the app and participants with mild AD dementia (n=6) spent a mean of 104 (SD 38.67, 95% CI 73.05-134.94) days, with 27.39 (SD 6.97, 95% CI 21.82-32.97) minutes per day on the app.

#### Performance on Constant Therapy Tasks

To assess the preliminary efficacy of the intervention, we analyzed the accuracy and latency scores of the Constant Therapy group at the start and end of the 24-week Constant Therapy training (Table 2). Table 2 shows the task scores that improved over 24 weeks. In the Multimedia Appendices 2 and 3 we also present the by-participant task progression over the 24-week intervention period (Multimedia Appendix 1) and by participant improvements and decrements across the individual tasks (Multimedia Appendix 2).

**Table 2:** Constant Therapy tasks performance over 24 weeks.

Task		Start, median (IQR)	End, median (IQR)	*z*-score	*P* value
**Accuracy**					
	Cumulative	0.89 (0.04)	0.91 (0.02)	2.8	.005
	Addition	0.93 (0.08)	0.98 (0.05)	2.49	.01
	Environmental sound matching	0.86 (0.14)	0.95 (0.07)	2.55	.01
	Picture N-back memory	0.88 (0.18)	0.95 (0.09)	2.93	.02
	Written word matching	0.87 (0.07)	0.90 (0.06)	1.99	.047
**Latency**					
	Cumulative	25.78 (11.57)	21.96 (9.6)	−2.8	.005
	Addition	17.88 (6.67)	12.37 (4.34)	−2.8	.005
	Environmental sound matching	33.52 (16.13)	25.45 (10.4)	−2.67	.008
	Picture N-back memory	25.75 (3.44)	24.3 (3.12)	−2.8	.005
	Auditory Command	34.8 (27.51)	30.02 (10.22)	−2.8	.005
	Calendar reading	19.27 (15.99)	15.74 (14.31)	−2.2	.03
	Clock reading	6.22 (3.27)	4.82 (5.09)	−2.07	.04
	Currency	25.85 (19.23)	21.01 (11.81)	−2.39	.02
	Picture matching	50.28 (28.03)	47.41 (30.91)	−2.39	.02
	Spoken Word matching	72.58 (68.21)	68.86 (56.2)	−1.99	.047
	Playing-card slapjack	22.8 (1.75)	21.26 (0.99)	−2.07	.04
	Symbol matching	18.79 (10.87)	15.56 (10.31)	−2.08	.005
	Voicemail	29.99 (6.64)	28.3 (4.9)	−1.99	.046
	Word problem	62.52 (59.5)	49.68 (41.25)	−2.5	.01

#### Preliminary Efficacy of Constant Therapy on Clinical Variables

As stated in our analytic plan, we compared changes from week 0 to week 6 using the Mann-Whitney test for all RBANS subtests. When computing Mann-Whitney, changes in RBANS coding scores from week 0 to week 6 were significantly different between the 2 condition groups (*U*=80.00; *z*=2.867; *P*=.003), and the Constant Therapy group, with a median of 6.5 (IQR 5.75), had a larger improvement in coding scores compared with the active control group, with a median of 1 (IQR 3) after the first 6 weeks in the study. No other differences were observed between the RBANS subscores between weeks 0 and 6 (*P*>.139).

Next, since normally distributed, we also conducted a repeated measures ANOVA to explore changes in the coding scores over the full 24 weeks. We found a main effect of time (*F*_4,32_=4.34; *P=*.006; η^2^=0.35). Coding score at week 0 (mean 24.71, SD 9.08; SE 3.18) was higher than (1) week 6 (mean 29.02, SD 10.60; SE 3.32; *P=*.002); (2) week 12 (mean 29.36, SD 11.27; SE 3.46; *P=*.01); (3) week 18 (mean 28.69, SD 11.73; SE 3.50; *P=*.02); and (4) week 24 (mean 27.05, SD 11.47; SE 3.29; *P=*.02). No main effect of group was found (*F*_1,8_=3.22; *P=*.11; η^2^=0.29). There was an interaction between group and time (*F*_4,32_=4.06; *P=*.009; η^2^=0.34). The Constant Therapy group performed better at week 6 (mean 33.60, SD 6.26; SE 3.63; *P=*.001), week 12 (mean 33.89, SD 7.59; SE 3.79; *P=*.006), week 18 (mean 35.33, SD 7.70; SE 3.84; *P=*.005) and week 24 (mean 35.62, SD 8.18; SE 3.60; *P*<.001) compared with week 0 (mean 27.7, SD 7.00; SE 3.48). No significant differences were observed in the active control group over time.

**Figure 3:**
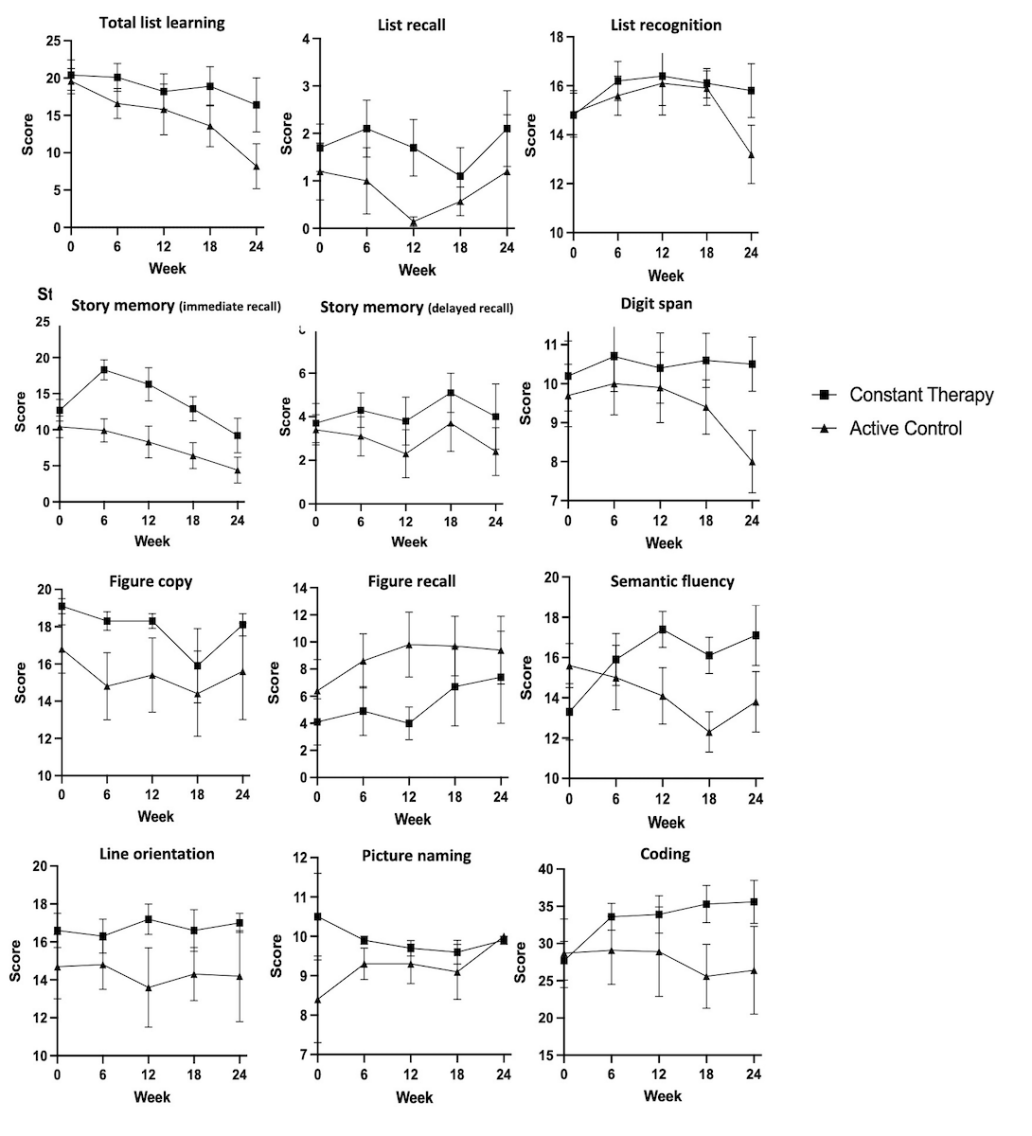
Mean and SE for the 12 Repeatable Battery for the Assessment of Neuropsychological Status subtests over 24 weeks.

The score changes across the first 24 weeks in the Constant Therapy and active control groups in each RBANS subtest administered are presented in Figure 3.

## Discussion

### Principal Findings

Our study aimed to examine the feasibility of home-based, self-administered, and long-term individualized cognitive training using Constant Therapy in patients with AD. In addition, as a secondary aim, we sought to evaluate the preliminary efficacy of the Constant Therapy training program on neuropsychological performance. Overall, this feasibility study aims to inform the development of a future RCT.

We predicted that patients with AD would adhere to the training over a 24-week period using the Constant Therapy app. Consistent with this prediction, we found that long-term use of the Constant Therapy program was feasible in a patient population with AD, as shown by the rate of adherence (80%) and use of the program (average of 121 out of 168 days for 32 minutes daily) over 24 weeks. In comparison, the participants assigned to the active control group had a 55% adherence rate to the study at 24 weeks. Our adherence rates are comparable with those of other RCTs testing computerized cognitive training in older adults with cognitive impairment (eg, 77% of patients with MCI in a 6-week period study adhered to the intervention and 76% adhered to the control sessions [44]), in patients with other neurological diseases (eg, 76% adherence to an 18-week training period in patients with Huntington disease [45]), and in more heterogeneous cohorts (eg, 83% adherence over a 24-week training period in patients with a variety of neurological and psychiatric diseases [46]).

Adherence rates dropped during the second optional part of the study, both in the Constant Therapy group (40%), who voluntarily continued the training, and the active control group (22%), who could begin the Constant Therapy training at 24 weeks. Due to the drop in adherence and loss of power between the first 24 weeks and the second 24 weeks, we did not examine the data collected between weeks 24 and 48. Although the data were not assessed, low adherence to the second portion of the study is helpful in informing the design and length of future RCTs. A future RCT should focus on testing the efficacy of a 24-week intervention training program.

The overall sustained performance on computerized tasks shows that the individualized training approach modeled by Constant Therapy is appropriate for a population with AD. Furthermore, not only did patients sustain their performance in the tasks but they also displayed improvements in performance that are important to report for future RCT studies. Specifically, patients performed more accurately over time in the task training domains of visual and auditory memory, attention, and arithmetic. Latency also improved in tasks related to visuospatial processing, visual and auditory memory, attention, quantitative reasoning, and arithmetic skills. Faster reaction time in the Constant Therapy tasks may suggest improvement in processing speed, as well as improved adaptability to computerized tasks. Faster reaction time not paired with improved accuracy may also represent increased disinhibition while completing the task. These positive findings suggest that the 24-week intervention program is feasible and underscore the need for future larger studies to test the effectiveness of Constant Therapy as a long-term training program for patients with AD dementia.

We also predicted that gains made using the Constant Therapy program would transfer to neuropsychological test performance. We monitored changes in performance by administering the RBANS every 6 weeks and the Constant Therapy group showed an improvement in coding abilities over time. Coding performance improved during the first 6 weeks and then remained stable over the remaining 18 weeks. Although we experienced a higher drop in adherence in the active control group compared with the Constant Therapy group over the first 24 weeks (which ultimately limits our ability to interpret findings pertaining to this prediction), the observed change in performance between the Constant Therapy and active control groups on the RBANS subtest of coding, despite the small numbers of participants, indicate that, to some extent, gains made during Constant Therapy have the potential to transfer onto neuropsychological test performance.

Coding was the only measure that showed an improvement in performance over time when using Constant Therapy. Coding is often used as a measure of executive function in the neuropsychological assessment of patients with dementia [47,48]. This pattern of results is consistent with previous literature showing that computerized cognitive training may lead to improvements in cognitive performance in the executive function domain [49]. While exploratory in nature, these results support previous work showing that computerized cognitive training programs have the potential to improve performance in neuropsychological tests and help mitigate cognitive decline in older adults with AD [15]. The results of the outcome measures in this study highlight the usefulness of frequent neuropsychological monitoring when designing long-term intervention studies. Future research examining the feasibility of long-term individualized computerized programs in similar populations could incorporate frequent neuropsychological tests into their designs to better assess the impact of these interventions on cognition.

Long-term computerized cognitive trainings (eg, 24 weeks) have shown to be effective in healthy older adults [16]. Our primary aim was to examine whether they are feasible in the population with AD using the Constant Therapy platform. Although several studies have investigated computerized cognitive training in patients with AD, most have consisted of intervention periods that do not exceed 8 weeks [44]. In addition, until now, Constant Therapy has most often been used for the rehabilitation of language or cognitive deficits caused by stroke or TBI [24,50] and has not yet been tested in patients in AD. This study indicates that the Constant Therapy home-based individualized program is feasible for 24 weeks and may be a beneficial tool for patients with AD. These findings build on recent evidence showing that patients with dementia demonstrate improvement in global cognitive function when provided with individualized cognitive training in both a traditional or remote clinical setting [51].

Furthermore, our data indicate that the Constant Therapy program can be feasible in both the MCI and mild dementia stages of AD, providing supporting evidence that the individualized progression in task difficulty and the length of the intervention were acceptable to patients given their adherence to the platform. A future larger RCT is needed to examine how computerized training may impact cognition and function in the daily lives of patients with AD. Finally, we note that the COVID-19 pandemic has posed new challenges to the feasibility of in-person cognitive training. Thus, now more than ever, the investigation of home-based, self-administered computerized platforms is essential to assist older adults with AD and related cognitive disorders [52].

### Limitations

This study tests the feasibility and attempts to test the preliminary efficacy of individualized, long-term, home-based computerized cognitive therapy in patients with AD. A limitation of our study is that we could not examine the data collected from weeks 24 to 48 due to the low rate of participation beyond 24 weeks. Nevertheless, the training length we examined (24 weeks) is still a valuable strength of our study, as it is a longer training period than that commonly tested in patients with AD. Finding new ways to actively involve caregivers in the program as a source of support throughout the intervention may help increase overall adherence over long periods. In addition, the drop in adherence over time in the active control group was greater than that in the Constant Therapy group. We believe that the use of paper-and-pencil games might have influenced retention in the active control group. While previous studies have shown no significant difference between the use of paper and computerized active control tasks on training effect [53], most of the work showing positive benefits from cognitive training used a computerized active control condition [16]. Computerized tasks are more interactive and entertaining, which could result in increased levels of motivation for the active control group [12].

Some limitations due to practical implementation considerations, the study design, and the patient population must be acknowledged. First, study staff members administering the batteries were not blinded, and the total sample size was 19. While this choice enabled our staff to answer questions related to the app during weekly check-ins if needed, this study feature, in combination with the small number of participants that engaged with the app, limited the conclusions. As such, we consider the preliminary efficacy findings to be exploratory. Nevertheless, our findings, especially regarding feasibility, help inform the suitability of training for larger trials in the future. In addition, we administered only the limited testing battery (ie, RBANS) at each 6-week interval and not surveys measuring metamemory, quality of life, and caregiver burden, for example. While this choice allowed us to reduce participant burden (because participants only had to complete approximately 40 minutes of testing), it is unknown whether engagement with the app benefited some other dimension of the participants’ and caregivers’ life experiences other than patient cognition. Furthermore, acceptability measures, such as satisfaction levels regarding the study in general, its individual components, or open-ended questions to collect feedback, were not assessed. Future RCTs should assess acceptability measures to help improve users’ experiences and overall adherence to interventions. In addition, larger RCTs should monitor and report the time point of the study where each participant dropped out. This categorization is important to examine the efficacy of the training in relation to the frequency and length of the assessments over the study period.

Finally, we acknowledge that we did not formally measure why participants stopped engaging with the app. However, in our discussions with the study coordinator, some participants may have experienced some difficulty with the auditory tasks, especially when the level of difficulty increased. It is possible that hearing difficulty might have impacted adherence to the training program as a whole and interfered with the effectiveness of the intervention on cognitive function.

Finally, due to the homogeneity of our sample, we cannot make any conclusions about the feasibility of the Constant Therapy training in other demographic groups. Of note, a factor contributing to this limitation is that our study lacked representation of women, and racially and ethnically minoritized groups.

The implications of this limitation are well acknowledged in such cognitive rehabilitation studies. For example, motivation, initial cognitive ability, and income level have the potential to affect cognitive interventions [54]. Furthermore, the use of technology may be differentially accessed and used based on factors such as one’s level of education [55]. While published work has shown that Constant Therapy rehabilitation training is equally feasible for individuals with speech language disorders who live in different geographic areas (urban vs rural) [26], future RCT studies should further explore the role of different demographic factors, including ethnicity, socioeconomic status, education, and gender, on the feasibility of Constant Therapy in the AD population.

### Conclusions

Home-based, self-administered, computerized cognitive trainings are a potential tool to help sustain cognitive function in patients with dementia [15,51]. Our study aimed to test the feasibility and preliminary efficacy of the Constant Therapy platform for patients with AD. Despite some of the challenging aspects of long-term intervention studies, our data show that Constant Therapy is a feasible platform for patients with AD for an intervention period of 24 weeks. Our findings support previous evidence that home-based self-administered Constant Therapy programs are a feasible alternative to in-person supervised cognitive training programs [25]. The patients with AD in our study engaged in Constant Therapy tasks and improved their performance over time. An exploratory analysis also showed promising changes in the RBANS coding subtest, which is a measure of executive function.

Future trials are necessary to investigate the efficacy of Constant Therapy training over a 24-week period on cognition and daily life function and to test adherence to longer intervention periods, such as 48 weeks. Thus, while long-term individualized Constant Therapy is feasible in patients with AD, more research is needed to explore its benefits and the factors that can influence its efficacy.

## Appendix

Multimedia Appendix 1 Constant Therapy use over the first 24 weeks.

Multimedia Appendix 2 Constant Therapy task levels completion over 24 weeks.

Multimedia Appendix 3 Constant Therapy tasks performance over 24 weeks.

Multimedia Appendix 4 CONSORT-EHEALTH checklist (V 1.6.1).

## Abbreviations

AD: Alzheimer disease
MCI: mild cognitive impairment
RBANS: Repeatable Battery for the Assessment of Neuropsychological Status
RCT: randomized controlled trial
TBI: traumatic brain injury
