# Supplementary figures and images for: Home-Based Electronic Cognitive Therapy in Patients With Alzheimer Disease: Feasibility Randomized Controlled Trial
Source: JMIR Form Res. 2022 Sep 12;6(9):e34450. doi: 10.2196/34450 (PMC9513684; doi:10.2196/34450)

**Multimedia Appendix 1.** Constant Therapy use over the first 24 weeks.


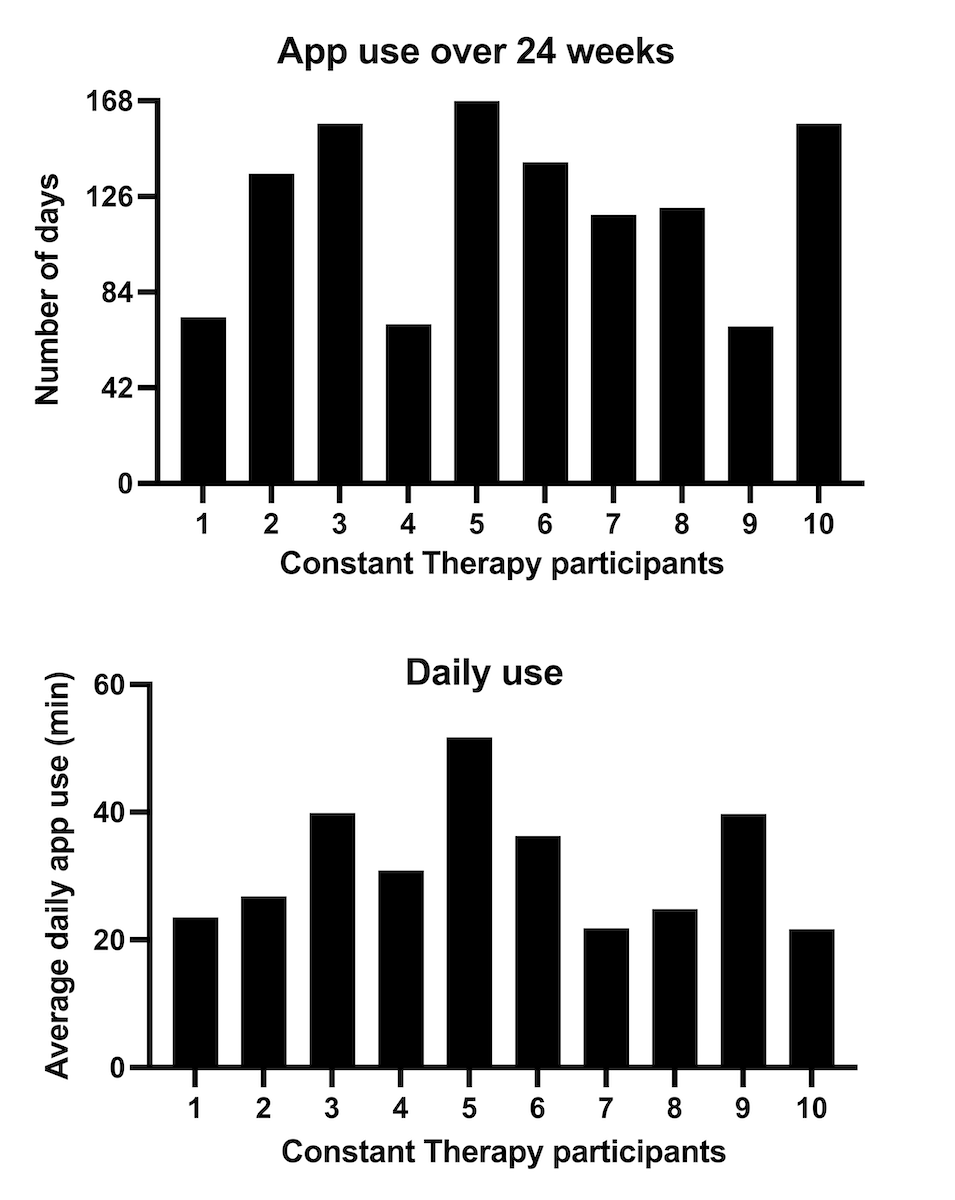

Supplement: Multimedia Appendix 1 [file formative_v6i9e34450_app1.docx]
