# Supplementary material for: Home-Based Electronic Cognitive Therapy in Patients With Alzheimer Disease: Feasibility Randomized Controlled Trial
Source: JMIR Form Res. 2022 Sep 12;6(9):e34450. doi: 10.2196/34450 (PMC9513684; doi:10.2196/34450)
Supplement: Multimedia Appendix 2 [file formative_v6i9e34450_app2.docx]

| Cognitive domain and task type  **Multimedia Appendix 2.** Constant Therapy task levels completion over 24 weeks.  in | | Participant ID | | | | | | | | | |
| --- | --- | --- | --- | --- | --- | --- | --- | --- | --- | --- | --- |
|  | | 1 | 2 | 3 | 4 | 5 | 6 | 7 | 8 | 9 | 10 |
|  | | | | | | | | | | | |
| **Arithmetic** | | | | | | | | | | | |
|  | Addition |  |  |  |  |  |  |  |  |  |  |
|  | Multiplication |  |  |  |  |  |  |  |  |  |  |
|  | Subtraction |  |  |  |  |  |  |  |  |  |  |
|  | Division |  |  |  |  |  |  |  |  |  |  |
| **Auditory comprehension and auditory memory** | | | | | | | | | | | |
|  | Environmental sound matching |  |  |  |  |  |  |  |  |  |  |
|  | Spoken Word matching |  |  |  |  |  |  |  |  |  |  |
|  | Voicemail |  |  |  |  |  |  |  |  |  |  |
|  | Auditory Command |  |  |  |  |  |  |  |  |  |  |
| **Visual processing, visual memory and attention** | | | | | | | | | | | |
|  | Calendar reading |  |  |  |  |  |  |  |  |  |  |
|  | Clock math |  |  |  |  |  |  |  |  |  |  |
|  | Clock reading |  |  |  |  |  |  |  |  |  |  |
|  | Map reading |  |  |  |  |  |  |  |  |  |  |
|  | Mental rotation |  |  |  |  |  |  |  |  |  |  |
|  | Pattern recreation |  |  |  |  |  |  |  |  |  |  |
|  | Picture matching |  |  |  |  |  |  |  |  |  |  |
|  | Face matching |  |  |  |  |  |  |  |  |  |  |
|  | Picture N-back memory |  |  |  |  |  |  |  |  |  |  |
|  | Playing-card slapjack |  |  |  |  |  |  |  |  |  |  |
|  | Symbol matching |  |  |  |  |  |  |  |  |  |  |
|  | Written word matching |  |  |  |  |  |  |  |  |  |  |
|  | Flanker |  |  |  |  |  |  |  |  |  |  |
| **Quantitative reasoning** | | | | | | | | | | | |
|  | Currency |  |  |  |  |  |  |  |  |  |  |
|  | Functional math |  |  |  |  |  |  |  |  |  |  |
|  | Number pattern |  |  |  |  |  |  |  |  |  |  |
|  | Word problem |  |  |  |  |  |  |  |  |  |  |

| Levels completed | |
| --- | --- |
| 0 |  |
| 1 |  |
| 2 |  |
| 3 |  |
| 4 |  |
| 5 |  |
| > 5 |  |
