# Supplementary material for: Home-Based Electronic Cognitive Therapy in Patients With Alzheimer Disease: Feasibility Randomized Controlled Trial
Source: JMIR Form Res. 2022 Sep 12;6(9):e34450. doi: 10.2196/34450 (PMC9513684; doi:10.2196/34450)
Supplement: Multimedia Appendix 3 [file formative_v6i9e34450_app3.docx]

**Multimedia Appendix 3.** Constant Therapy tasks performance over 24 weeks.

The following tables (a-d) display the progress made by each participant in the Constant Therapy group throughout the first 24-week period for each task type and level. Changes in accuracy and latency between the first and last 10 trials are displayed [24]. Six color codes have been assigned depending on the type of progress that was made through the 24-week intervention period.

We classified performance change in the Constant Therapy tasks into six different categories based on direction of change and latency and/or accuracy changes. Accuracy improvement was determined when accuracy scores increased between the average of the first 10 and last 10 trials. Accuracy worsening was determined when accuracy score decreased between the average of the first 10 and the last 10 trials. Latency improvement was determined when latency scores decreased between the average of the first 10 and the last 10 trials. Latency worsening was determined when latency scores increased between the average of the first 10 and the last 10 trials. Accuracy or latency scores were determined stable if the average of the 10 and the last 10 trials was the same.

1. Latency and accuracy changes in the arithmetic domain.

|  | task not administered |
| --- | --- |
|  | latency and accuracy improved |
|  | one improved, one stable |
|  | one improved, one worsened |
|  | one worsened, one stable |
|  | both worsened |
|  | both stable |

|  |  |  | Participant ID | | | | | | | | | |  |
| --- | --- | --- | --- | --- | --- | --- | --- | --- | --- | --- | --- | --- | --- |
| Cognitive Domain | Task Type | Task Level | 1 | 2 | 3 | 4 | 5 | 6 | 7 | 8 | 9 | 10 | Total |
| Arithmetic | Addition | 1 |  |  |  |  |  |  |  |  |  |  |  |
|  |  | 2 |  |  |  |  |  |  |  |  |  |  |  |
|  |  | 3 |  |  |  |  |  |  |  |  |  |  |  |
|  |  | 4 |  |  |  |  |  |  |  |  |  |  |  |
|  |  | 5 |  |  |  |  |  |  |  |  |  |  |  |
|  | Multiplication | 1 |  |  |  |  |  |  |  |  |  |  |  |
|  |  | 2 |  |  |  |  |  |  |  |  |  |  |  |
|  |  | 3 |  |  |  |  |  |  |  |  |  |  |  |
|  |  | 4 |  |  |  |  |  |  |  |  |  |  |  |
|  |  | 5 |  |  |  |  |  |  |  |  |  |  |  |
|  | Subtraction | 1 |  |  |  |  |  |  |  |  |  |  |  |
|  |  | 2 |  |  |  |  |  |  |  |  |  |  |  |
|  |  | 3 |  |  |  |  |  |  |  |  |  |  |  |
|  |  | 4 |  |  |  |  |  |  |  |  |  |  |  |
|  |  | 5 |  |  |  |  |  |  |  |  |  |  |  |
|  | Division | 1 |  |  |  |  |  |  |  |  |  |  |  |
|  |  | 2 |  |  |  |  |  |  |  |  |  |  |  |
|  |  | 3 |  |  |  |  |  |  |  |  |  |  |  |
|  |  | 4 |  |  |  |  |  |  |  |  |  |  |  |
|  |  | 5 |  |  |  |  |  |  |  |  |  |  |  |

1. Latency and accuracy changes in the auditory domain.

|  | task not administered |
| --- | --- |
|  | latency and accuracy improved |
|  | one improved, one stable |
|  | one improved, one worsened |
|  | one worsened, one stable |
|  | both worsened |
|  | both stable |

|  |  |  | Participant ID | | | | | | | | | |  | |
| --- | --- | --- | --- | --- | --- | --- | --- | --- | --- | --- | --- | --- | --- | --- |
| Cognitive Domain | Task Type | Task Level | 1 | 2 | 3 | 4 | 5 | 6 | 7 | 8 | 9 | 10 | Total |  |
| Auditory comprehension and auditory memory | Environmental Sound Matching | 1 |  |  |  |  |  |  |  |  |  |  |  |  |
|  |  | 2 |  |  |  |  |  |  |  |  |  |  |  |  |
|  |  | 3 |  |  |  |  |  |  |  |  |  |  |  |  |
|  | Spoken Word Matching | 1 |  |  |  |  |  |  |  |  |  |  |  |  |
|  |  | 2 |  |  |  |  |  |  |  |  |  |  |  |  |
|  |  | 3 |  |  |  |  |  |  |  |  |  |  |  |  |
|  |  | 4 |  |  |  |  |  |  |  |  |  |  |  |  |
|  |  | 5 |  |  |  |  |  |  |  |  |  |  |  |  |
|  | Voicemail | 2 |  |  |  |  |  |  |  |  |  |  |  |  |
|  | Auditory Command | 1 |  |  |  |  |  |  |  |  |  |  |  |  |
|  |  | 2 |  |  |  |  |  |  |  |  |  |  |  |  |
|  |  | 3 |  |  |  |  |  |  |  |  |  |  |  |  |
|  |  | 4 |  |  |  |  |  |  |  |  |  |  |  |  |
|  |  | 5 |  |  |  |  |  |  |  |  |  |  |  |  |

1. Latency and accuracy changes in the visual domain.

|  | task not administered |
| --- | --- |
|  | latency and accuracy improved |
|  | one improved, one stable |
|  | one improved, one worsened |
|  | one worsened, one stable |
|  | both worsened |
|  | both stable |

|  |  |  | Participant ID | | | | | | | | | |  | |
| --- | --- | --- | --- | --- | --- | --- | --- | --- | --- | --- | --- | --- | --- | --- |
| Cognitive Domain | Task Type | Task Level | 1 | 2 | 3 | 4 | 5 | 6 | 7 | 8 | 9 | 10 | Total |  |
| Visual processing, visual memory, and attention | Calendar Reading | 1 |  |  |  |  |  |  |  |  |  |  |  |  |
|  |  | 2 |  |  |  |  |  |  |  |  |  |  |  |  |
|  | Clock Math | 1 |  |  |  |  |  |  |  |  |  |  |  |  |
|  |  | 2 |  |  |  |  |  |  |  |  |  |  |  |  |
|  |  | 3 |  |  |  |  |  |  |  |  |  |  |  |  |
|  | Clock Reading | 1 |  |  |  |  |  |  |  |  |  |  |  |  |
|  |  | 2 |  |  |  |  |  |  |  |  |  |  |  |  |
|  | Map Reading | 1 |  |  |  |  |  |  |  |  |  |  |  |  |
|  |  | 2 |  |  |  |  |  |  |  |  |  |  |  |  |
|  |  | 3 |  |  |  |  |  |  |  |  |  |  |  |  |
|  | Mental Rotation | 1 |  |  |  |  |  |  |  |  |  |  |  |  |
|  | Pattern Recreation | 1 |  |  |  |  |  |  |  |  |  |  |  |  |
|  |  | 2 |  |  |  |  |  |  |  |  |  |  |  |  |
|  |  | 3 |  |  |  |  |  |  |  |  |  |  |  |  |
|  |  | 4 |  |  |  |  |  |  |  |  |  |  |  |  |
|  |  | 5 |  |  |  |  |  |  |  |  |  |  |  |  |
|  |  | 6 |  |  |  |  |  |  |  |  |  |  |  |  |
|  | Picture Matching | 1 |  |  |  |  |  |  |  |  |  |  |  |  |
|  |  | 2 |  |  |  |  |  |  |  |  |  |  |  |  |
|  |  | 3 |  |  |  |  |  |  |  |  |  |  |  |  |
|  |  | 4 |  |  |  |  |  |  |  |  |  |  |  |  |
|  | Face Matching | 1 |  |  |  |  |  |  |  |  |  |  |  |  |
|  |  | 2 |  |  |  |  |  |  |  |  |  |  |  |  |
|  |  | 3 |  |  |  |  |  |  |  |  |  |  |  |  |
|  |  | 4 |  |  |  |  |  |  |  |  |  |  |  |  |
|  | Picture N-Back Memory | 1 |  |  |  |  |  |  |  |  |  |  |  |  |
|  |  | 2 |  |  |  |  |  |  |  |  |  |  |  |  |
|  | Playing Card Slapjack | 1 |  |  |  |  |  |  |  |  |  |  |  |  |
|  | Symbol Matching | 1 |  |  |  |  |  |  |  |  |  |  |  |  |
|  |  | 2 |  |  |  |  |  |  |  |  |  |  |  |  |
|  |  | 3 |  |  |  |  |  |  |  |  |  |  |  |  |
|  |  | 4 |  |  |  |  |  |  |  |  |  |  |  |  |
|  |  | 5 |  |  |  |  |  |  |  |  |  |  |  |  |
|  |  | 6 |  |  |  |  |  |  |  |  |  |  |  |  |
|  |  |  |  |  |  |  |  |  |  |  |  |  |  |  |
|  |  | 7 |  |  |  |  |  |  |  |  |  |  |  |  |
|  |  | 8 |  |  |  |  |  |  |  |  |  |  |  |  |
|  |  | 9 |  |  |  |  |  |  |  |  |  |  |  |  |
|  |  | 10 |  |  |  |  |  |  |  |  |  |  |  |  |
|  | Written Word Matching | 1 |  |  |  |  |  |  |  |  |  |  |  |  |
|  |  | 2 |  |  |  |  |  |  |  |  |  |  |  |  |
|  |  | 3 |  |  |  |  |  |  |  |  |  |  |  |  |
|  |  | 4 |  |  |  |  |  |  |  |  |  |  |  |  |
|  | Flanker | 1 |  |  |  |  |  |  |  |  |  |  |  |  |

1. Latency and accuracy changes in the quantitative reasoning domain.

|  | task not administered |
| --- | --- |
|  | latency and accuracy improved |
|  | one improved, one stable |
|  | one improved, one worsened |
|  | one worsened, one stable |
|  | both worsened |
|  | both stable |

|  |  |  | Participant ID | | | | | | | | | |  |
| --- | --- | --- | --- | --- | --- | --- | --- | --- | --- | --- | --- | --- | --- |
| Cognitive Domain | Task Type | Task Level | 1 | 2 | 3 | 4 | 5 | 6 | 7 | 8 | 9 | 10 | Total |
| Quantitative Reasoning | Currency | 1 |  |  |  |  |  |  |  |  |  |  |  |
|  |  | 2 |  |  |  |  |  |  |  |  |  |  |  |
|  |  | 3 |  |  |  |  |  |  |  |  |  |  |  |
|  |  | 4 |  |  |  |  |  |  |  |  |  |  |  |
|  | Functional Math | 1 |  |  |  |  |  |  |  |  |  |  |  |
|  |  | 2 |  |  |  |  |  |  |  |  |  |  |  |
|  |  | 3 |  |  |  |  |  |  |  |  |  |  |  |
|  | Number Pattern | 1 |  |  |  |  |  |  |  |  |  |  |  |
|  |  | 2 |  |  |  |  |  |  |  |  |  |  |  |
|  |  | 3 |  |  |  |  |  |  |  |  |  |  |  |
|  |  | 4 |  |  |  |  |  |  |  |  |  |  |  |
|  |  | 5 |  |  |  |  |  |  |  |  |  |  |  |
|  | Word Problem | 1 |  |  |  |  |  |  |  |  |  |  |  |
|  |  | 2 |  |  |  |  |  |  |  |  |  |  |  |
|  |  | 3 |  |  |  |  |  |  |  |  |  |  |  |
|  |  | 4 |  |  |  |  |  |  |  |  |  |  |  |
|  |  | 5 |  |  |  |  |  |  |  |  |  |  |  |
